# Chitosan Activated with Genipin: A Nontoxic Natural Carrier for Tannase Immobilization and Its Application in Enhancing Biological Activities of Tea Extract

**DOI:** 10.3390/md19030166

**Published:** 2021-03-19

**Authors:** Chi Wang, Pei-Xu Chen, Qiong Xiao, Qiu-Ming Yang, Hui-Fen Weng, Yong-Hui Zhang, An-Feng Xiao

**Affiliations:** 1 College of Food and Biological Engineering, Jimei University, Xiamen 361021, China; wangmou007@jmu.edu.cn (C.W.); chenxuxu1205@jmu.edu.cn (P.-X.C.); xiaoqiong129@jmu.edu.cn (Q.X.); yangqm@jmu.edu.cn (Q.-M.Y.); wenghuifen@jmu.edu.cn (H.-F.W.); 2 Fujian Provincial Engineering Technology Research Center of Marine Functional Food, Xiamen 361021, China; 3 Xiamen Key Laboratory of Marine Functional Food, Xiamen 361021, China

**Keywords:** chitosan, genipin, tannase, tea extract, antioxidant activity, α-amylase, α-glucosidase

## Abstract

In this work, a non-toxic chitosan-based carrier was constructed via genipin activation and applied for the immobilization of tannase. The immobilization carriers and immobilized tannase were characterized using Fourier transform infrared spectroscopy and thermogravimetric analysis. Activation conditions (genipin concentration, activation temperature, activation pH and activation time) and immobilizations conditions (enzyme amount, immobilization time, immobilization temperature, immobilization pH, and shaking speed) were optimized. The activity and activity recovery rate of the immobilized tannase prepared using optimal activation and immobilization conditions reached 29.2 U/g and 53.6%, respectively. The immobilized tannase exhibited better environmental adaptability and stability. The immobilized tannase retained 20.1% of the initial activity after 12 cycles and retained 81.12% of residual activity after 30 days storage. The catechins composition analysis of tea extract indicated that the concentration of non-ester-type catechins, EGC and EC, were increased by 1758% and 807% after enzymatic treatment. Biological activity studies of tea extract revealed that tea extract treated with the immobilized tannase possessed higher antioxidant activity, higher inhibitory effect on α-amylase, and lower inhibitory effect on α-glucosidase. Our results demonstrate that chitosan activated with genipin could be an effective non-toxic carrier for tannase immobilization and enhancing biological activities of tea extract.

## 1. Introduction

Green tea extracts, which constitutes approximately 30% of the weight of the tea leaves, play a key role because they are rich in polyphenols and have various biological activities [1,2]. The predominant forms of tea polyphenols include catechins and other flavanol derivatives [3], such as epigallocatechin gallate (EGCG), epigallocatechin (EGC), epicatechin gallate (ECG), and epicatechin (EC). Despite their excellent biological activities, catechins are major contributors to the astringent and bitter taste of green tea infusions [4]. In addition, polyphenol complexation is the main driver of tea paste formation and influenced by the quantities of galloyl and hydroxyl groups in polyphenols [5].

Tannase (Tannase, EC 3.1.1.20) is a hydrolase mainly derived from microorganism such as *Aspergillus* [6,7], *Penicillium* [8,9], and *Kluyveromyces* [10]. The ester bonds and carboxyphenolic acid bonds in polyphenol substances can be hydrolyzed by tannase to produce gallic acid and corresponding alcohols. Tannase was utilized to cleave the ester bonds of certain tannins in tea infusion and thereby prevented the complex formation of tannins with caffeine and increased the stability of tea infusion [11]. Ester-type catechins (EGCG and ECG) [12] in tea infusion could be hydrolyzed into non-ester-type catechins (EGC and EC), which improved the sweet aftertaste of tea infusion since EGC and EC are the main contributors for sweet aftertaste. Tannase treatment was also shown to increase polyphenol recovery rate [13,14] in tea extraction process due to the degradation due to its hydrolysis toward the diferulic acid dimer in the plant cell wall. The biological activities of tannase-treated tea extracts, such as antioxidant activity and inhibition effect on digestive enzymes, are also improved [15].

Despite the advantages of the enzymatic treatment of tea extracts, particularly high catalytic efficiency, mild reaction conditions, and low degree of contamination, it has the disadvantages of insufficient stability and inability to recycle, which make the process costly and difficult to implement [16,17]. Enzyme immobilization is a suitable approach to resolve this application problem. Enzyme immobilization normally utilized solid materials to restrain enzymes to a certain space for catalytic reaction, which can be recycled to reduce catalyst cost [18]. Immobilizing enzymes with proper immobilization strategy can greatly improve enzymatic properties and catalytic performance, including activity, stability, adaptability, specificity, selectivity, reduce inhibitions, etc. [19,20,21]. Many approaches for tannase immobilization have been proposed, for example, the use of carboxyl-functionalized Fe_3_O_4_ nanoparticles [22], chitin-alginate composites [23], and chitosan [24,25,26].

Chitosan an N-deacetylated derivative of chitin, mainly extracted from shells of marine crustaceans (crabs, shrimps, lobsters, etc.). Chitosan has unique chemical and biological properties. In its linear polyglucosamine chains of high molecular weight, chitosan has active amino and hydroxyl groups for easy chemical modification [27,28]. Moreover, the existence of amino groups makes chitosan a cationic polyelectrolyte (pKa ≈ 6.5). Thus, chitosan’s high solubility in acidic solutions and its aggregation with polyanions give it excellent glue-forming properties [29]. Owing to its nontoxicity, biodegradability properties, and biocompatibility, it has been widely used as an excellent natural material in industrial and biomedical applications, such as for protein delivery [30], drug delivery [31], as a gene carrier [32], and for enzyme immobilization [33,34,35]. As in the above applications, the various forms that chitosan polymers take, such as beads/microspheres, microcapsules, fibers, membranes, coatings, sponges, and gels with different geometrical configurations, expand their use as carriers to support a large number of biomolecules or industrially relevant enzymes [36]. Despite the promising application of various forms of chitosan polymers as carriers for immobilized enzymes, they have relatively weak properties and poor mechanical stability [37,38]. Enzymes could be immobilized by chitosan via simple adsorption, mainly through weak forces between the substrate and the enzyme, such as electrostatic or hydrophobic interactions. However, this may lead to enzyme leakage during recycle. Glutaraldehyde was utilized as a versatile activation reagent or cross-linking reagent for enhancing the immobilization efficiency of chitosan. However, from a safety point of view, glutaraldehyde was shown to be corrosive, irritating, toxic to humans, and hazardous to aquatic organisms [39]. For these reasons, the use of safer cross-linking agents to effectively immobilize enzymes remains an ongoing challenge.

Genipin is a natural chemical compound that can be extracted from gardenia fruit [40,41]. A genipin molecule contains a glutaraldehyde group, which facilitates the cross-linking of genipin with proteins, collagen, gelatin, and chitosan. As a novel cross-linking reagent for substituting glutaraldehyde, genipin has the advantages of natural source, high cross-linking efficiency, and low cytotoxicity (approximately 1/10,000 [42] that of glutaraldehyde). To date, genipin has been used in the immobilizing keratinase [43], β-galactosidase [24,26], and lipase [44]. However, to the best of our knowledge, immobilizing tannase on genipin cross-linked chitosan (GP–CS) carriers and the effect of tannase immobilized on GP–CS (GP–CS–tannase) on the biological activities of tea extracts have not been explored.

In this study, we selected tannase from *Aspergillus oryzae* FJ0123 as a model to explore the potential use of GP–CS in enzyme immobilization and enhancing the biological activities of tea extracts. The effect of genipin activation conditions and immobilization conditions, such as genipin concentration, enzyme concentration, pH, temperature, and reaction time, were investigated in detail. Catechins composition and the biological activities of tea extracts prepared with GP–CS-tannase were determined, and the reusability of GP–CS-tannase was examined.

## 2. Results and Discussion

### 2.1. Determination of the Working Activation Conditions for Chitosan Beads

A preliminary research was carried out to compare the effects of glutaraldehyde and genipin on the effect of chitosan immobilized enzyme. The results were listed in the supporting material. As shown in Figure S1, although the recovery rate of GP–CS-tannase was 3.2% lower than that activated with glutaraldehyde, genipin still exhibited a competitive efficiency for tannase immobilization compared with glutaraldehyde. Therefore, it is worth to investigate genipin immobilization of tannase for non-toxic and safe subsequent production of tea extracts.

**Figure 1:**
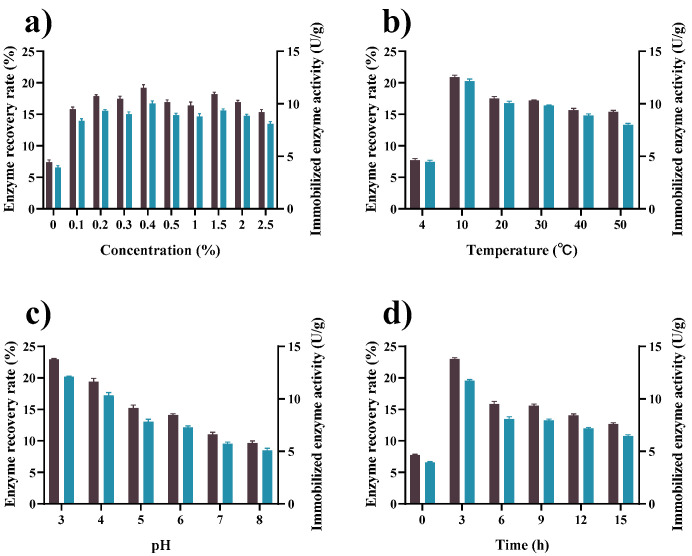
Effect of activation conditions: (**a**) genipin concentration (0–2.5%, *m*/*v*), (**b**) activation temperature (4–50 °C), (**c**) activation pH (citrate buffer at pH 3.0–6.0, and phosphate at pH 6.0–8.0), and (**d**) activation time (0–15 h) on the enzyme recovery rate (■) of immobilized tannase and enzyme activity (■); other immobilization conditions were carried out as in Section 3.2. After the activation step, excess genipin was washed off with 200 mL of deionized (DI) water, and GP–CS was used for immobilization. The activity of immobilized enzyme was determined under standard conditions (30 °C, citrate buffer at pH 5.0). All data represent the mean ± standard deviation of triplicate measurements.

The effect of activation conditions on the recovery of tannase activity after immobilization with GP–CS was examined. As can be seen in Figure 1a, the recovery of enzyme activity at this time reached 7.4% when the concentration of genipin in the system was 0. The activity was due to the physical adsorption and ion exchange [45,46,47] of chitosan on tannase, which was not easily eluted by the buffer. Enzyme activity recovery and specific enzyme activity were 19.2% and 10.0 U/g, respectively, when the amount of genipin was increased to 0.4 mg/mL. Continuous increase in the amount of genipin resulted in a decrease in enzyme activity because of genipin saturation and production of internal polymerization of genipin-genipin and genipin-chitosan, resulting in a decrease in the effective groups bound to the enzyme [43,48].

The effect of activation temperature on the enzyme recovery rate was studied. The result is shown in Figure 1b. The optimal temperature for chitosan activation was 10 °C, at which the highest immobilization efficiency (20.9%) was obtained. The result indicated that the activating reaction between genipin and chitosan exerted better at lower temperatures. When the activation temperature was raised to 50 °C, the recovery rate of tannase activity was only 15.4%, which was 75% of that at 10 °C.

The cross-linking reaction between chitosan and genipin had different reaction mechanisms at varied pH, which greatly influence the carrier activation process [49]. Under acidic and neutral conditions, the amino group of chitosan executes a nucleophilic attack on the alkene carbon atom at C-3, which then opens the dihydropyran ring, and the secondary amino group attacks the newly formed aldehyde group. In other words, genipin acts as a double aldehyde, but its condensation product is much more stable than glutaraldehyde. In the product, the shortchain of a condensed genipin acts as a cross-linking aldehyde. Under alkaline conditions, the ring-opening reaction of genipin results in the formation of intermediate aldehyde groups by facilitating the nucleophilic attack of hydroxyl groups in aqueous solutions, which then undergo aldol condensation reactions. The terminal aldehyde group on polymerized genipin undergoes a Schiff reaction with the chitosan amino group and forms a cross-linked network [49,50,51,52]. As shown in Figure 1c, the recovery rate of tannase activity reached 22.9% at pH 3, and the recovery rate decreased with increasing activation pH. When pH was adjusted to 8, the recovery rate of tannase activity was only 41.9% of that at pH 3. The high proton environment at a low pH provides suitable conditions for nucleophilic attack of primary amines on the C3 of genipin. Subsequently, the Schiff base reaction between the formed genipin aldehyde group and the enzyme protein occurs [53].

Figure 1d shows the effect of activation time on the immobilization process. When the activation time reached 3 h, the enzyme recovery rate and enzyme activity peaked at 22.9% and 11.7 U/g, respectively. Enzyme recovery rate and enzyme activity slowly decreased with increasing the activation time. An increase in activation time may intensify the internal polymerization of genipin–genipin, leading to a decrease in the effective ligation capacity of chitosan for tannase [54].

### 2.2. FTIR Analysis

**Figure 2:**
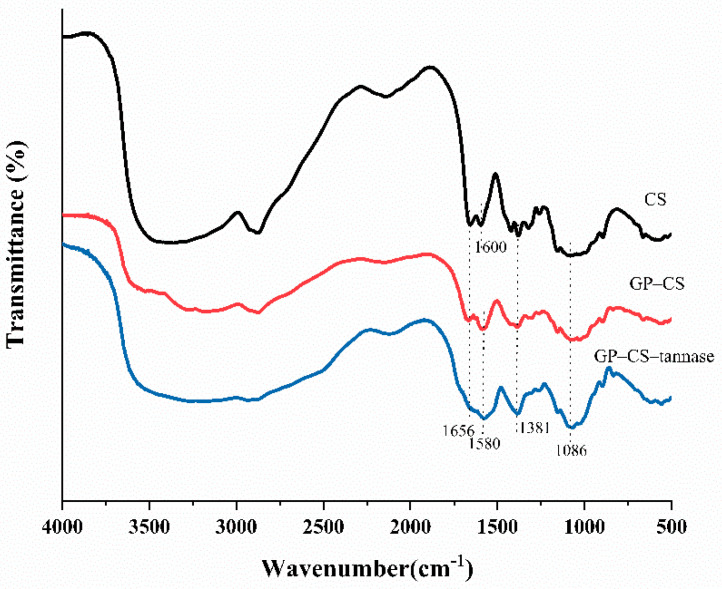
FTIR analysis of the CS, GP–CS, and GP–CS–tannase.

Figure 2 shows the FTIR spectra of CS, GP–CS, and GP–CS–tannase. The CS spectrum showed absorption peaks at 1656 and 1600 cm^−1^ [24], which indicated the N-H bending vibrations of the primary amine on the chitosan structure. Additionally, the symmetrical angular deformation peak characteristic of CH3 at 1381 cm^−1^ was observed [55]. The absorption spectrum from 1000 cm^−1^ and 1100 cm^−1^ was dominated by C-O and C-N stretching vibrations and C-C-N bending vibrations [56]. After cross-linking with genipin, amide II on GP–CS showed N-H deformation at 1580 cm^−1^. The deformation may have resulted from the reaction of genipin ester with hydroxyl and chitosan amino groups and produced secondary amides [56]. The peak at 1656 cm^−1^ was attributed to C=O stretching in the secondary amide. The spectrum of GP–CS–tannase has an amide band (1656 cm^−1^, 1580 cm^−1^) similar to that of GP–CS, since the mechanism involved in the cross-linking reaction in the presence of the enzyme was the same as that involved in the cross-linking reaction of chitosan particles (CS). The increase in characteristic band intensity may be due to an increase in available amino groups (from adsorption enzymes), which react with genipin, which promoted an increase in cross-linking groups, such as amide bonds [24]. These results indicated that the genipin groups attached to chitosan particles.

### 2.3. TGA Analysis

**Figure 3:**
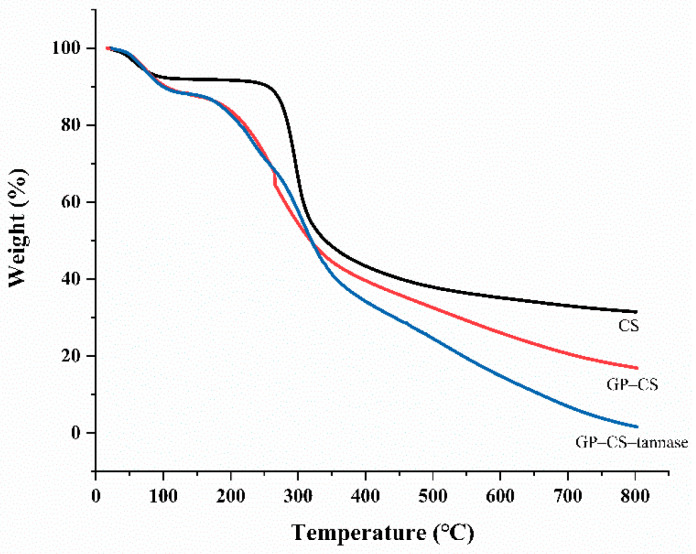
TGA analysis of CS, GP–CS, and GP–CS–tannase.

Figure 3 shows the TGA profiles of CS, GP–CS, and GP–CS–tannase. Weight loss in CS, GP–CS, and GP–CS–tannase occurred in three main temperature phases. Weight loss was observed in all samples when the temperature reached 100 °C due to the elimination of adsorbed water from the surface [24]. Chitosan particles (CS) showed a low weight loss rate of only 7.64% in this region compared with GP–CS (9.65%) and GP–CS–tannase (10.08%), indicating the low hydrophilicity of CS. Chitosan showed high thermal stability at temperatures of up to 240 °C and exhibited significant weight loss at temperatures ranging from 270 °C to 400 °C. This decomposition step can be ascribed to the complex dehydration, depolymerization, and pyrolytic decomposition of the polysaccharide structure (C-O-C and C-C bonds) [57], and to the evaporation and elimination of volatile products [24,58,59]. The weight loss rates from 180 °C to 400 °C for GP–CS pellets and GP–CS–tannase were 46.3% and 51.5%, respectively. In this temperature range, weight loss can be attributed to the weakening of chitosan structures after GP crosslinking. The total weight loss rates of GP–CS and GP–CS–tannase were 83.1% and 98.3%, respectively, as indicated by the TGA curve, suggesting that 15. 2% of tannase was immobilized on the surface of GP–CS. Furthermore, the TGA curves showed that the chitosan particles obtained were thermally stable in the temperature range used for most enzymatic reactions.

### 2.4. Determination of the Working Conditions for Tannase Immobilization

**Figure 4:**
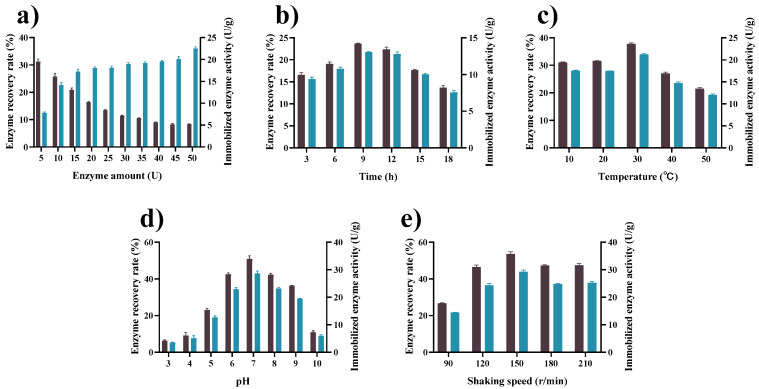
Effects of (**a**) enzyme amount (5–50 U), (**b**) immobilization time (3–18 h), (**c**) immobilization temperature (10–50 °C), (**d**) immobilization pH (Citrate buffer at pH 3.0–6.0, phosphate at pH 6.0–8.0, Tris-HCl buffer at pH 8.0–9.0, and Gly-NaOH buffer at pH 9.0–10.0), and (**e**) shaking speed (90–210 rpm/min) on the enzyme recovery rate (■) of immobilized tannase and the enzyme activity (■). Other immobilization conditions were carried out as in Section 3.3. The activity of immobilized enzyme was determined under standard conditions (30 °C, citrate buffer at pH 5.0). All data represent the mean ± standard deviation of triplicate measurements.

To obtain insights into the effect of immobilization conditions on the activity recovery of tannase after immobilization with GP–CS, we conducted a series of investigations. The effect of initial enzyme concentration on GP–CS–tannase activity is shown in Figure 4a. The highest recovery rate (31.15%) was achieved at a low tannase addition of 5 U. As the amount of tannase increased, the enzyme recovery rate gradually decreased, and the enzyme activity gradually increased. Considering the enzyme recovery rate and enzyme activity, we set the initial enzyme amount at 10 U. Under this condition, the recovery rate and enzyme activity were 25.7% and 14.1 U/g, respectively.

To determine the optimum reaction time for immobilization, we measured the enzyme recovery rate and activity of the immobilized enzymes at reaction times of 3, 6, 9, 12, 15, and 18 h. As shown in Figure 4b, when the reaction time was 9 h, enzyme activity and activity recovery reached their highest values (13.1 U/g and 23.7%, respectively). Enzyme activity and activity recovery gradually decreased with increasing immobilization time possibly due to the saturation of the reactive groups on the carrier, excessive physisorption, and the enzyme’s spatial resistance [60].

To access the effect of temperature on immobilization, we immobilized with GP–CS at 10 °C, 20 °C, 30 °C, 40 °C, and 50 °C. Figure 4c shows that the recovery of activity and immobilized enzyme activity reached their highest values (37.8% and 21.2 U/g, respectively) at 15 °C. With increasing temperature, immobilized tannase activity and recovery of activity gradually decreased probably due to the structural changes in GP–CS and tannase under high temperatures.

External pH influences the interaction between genipin and an enzyme, and the stability of enzymes [61]. To determine the optimum pH for the reaction between tannase and GP–CS, we set the pH values of the entire system to 3–10 and measured the enzyme recovery rate and activity of the immobilized enzymes at the end of the reaction. Figure 4d shows that the activity recovery and immobilized enzyme activity of GP–CS immobilized tannase peaked at pH 7 and had values of 50.8% and 28.5 U/g, respectively.

The optimum shaking speed (Figure 4e) for the immobilization reaction was determined by analyzing enzyme activity and activity recovery at different shaking speeds ranging from 90 rpm/min to 210 rpm/min. The optimum values (53.6% and 29.2 U/g, respectively) of enzyme activity recovery and enzyme activity of the immobilized enzyme were observed at 150 rpm/min. The effectiveness of immobilization decreased with increasing shaking speed. Therefore, the optimum oscillation speed for the immobilized enzyme was set at 150 rpm/min. The optimized immobilization conditions of GP–CS–tannase were as follows: initial enzyme amount, 10 U; temperature, 15 °C; pH, 7; shaking speed, 150 rpm/min. It is noteworthy that the optimal conditions resulted in 93.2% immobilization yield when the tannase activity was impaired probably due to aggregation, unfavorable protein conformational changes, or steric hindrance to the active site. The immobilization yield and activity recovery rate were similar to other reports about enzyme immobilization with activated chitosan beads.

### 2.5. Enzymatic Properties of Tannase and GP–CS–Tannase

**Figure 5:**
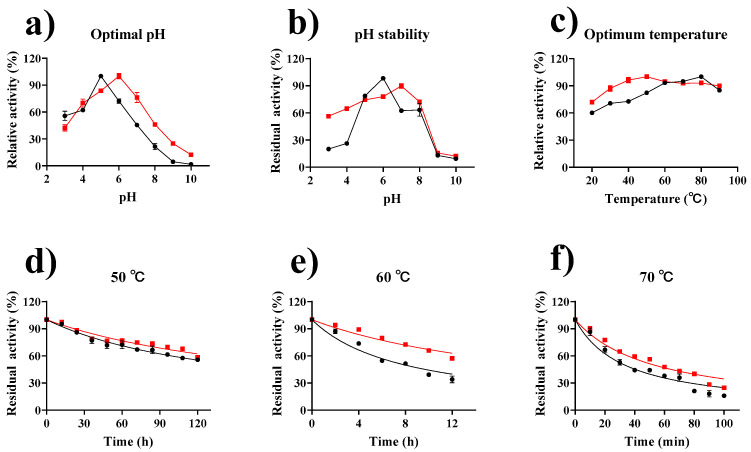
Effects of pH and temperature on the activity and stability of tannase (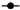) and GP–CS–tannase (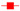). (**a**) The optimal pH For Tannase was determined by performing an activity assay with four different buffers (50 mM) at pH 3.0–10.0, citrate buffer at pH 3.0–6.0, phosphate at pH 6.0–8.0, Tris-HCl buffer at pH 8.0–9.0, and Gly-NaOH buffer at pH 9.0–10.0. (**b**) The pH stability of Tannase was determined during incubation at different pH values and 4 °C for 24 h. (**c**) The determination of the optimum temperatures for free and immobilized tannase at different temperatures and at pH 5.0 and 6.0. (**d**–**f**) The thermal stability of free and immobilized tannase was determined after incubation at 50 °C, 60 °C, and 70 °C. The half-life was obtained by fitting with GraphPad Prism software. All data represent the mean ± standard deviation of triplicate measurements.

The pH of the catalytic environment significantly affects the reaction catalyzed by an enzyme [62]. As can be seen in Figure 5a, the optimum pH of the immobilized tannase was 6 and shifted toward a neutral pH. In addition, the activity of GP–CS–tannase was higher than that of free tannase between pH 6.0 and 10.0, indicating that the immobilized tannase was less sensitive to environmental pH than free tannase. Tannase has a broader pH range of activity when immobilized than free enzymes [22,63]. Change in pH may be due to a reduction in the number of the positively charged groups of enzymes associated the carriers’ amino groups after immobilization. As a result, the enzymes become polyanionic. The pH stability of free tannase and immobilized tannase was assessed in the pH range of 3.0–10.0. As shown in Figure 5b, the immobilized tannase was more stable than the free tannase at pH 3–8. At pH ranging from 3 to 8, the discrepancy between the maximum and minimum values of the residual enzyme activity of the immobilized enzyme was only 33.4%, whereas the discrepancy reached 78.2% for the free enzyme. These results can be explained by the protective effects of the microenvironment of the immobilized carrier and the buffering effect of the carrier microenvironment; these effects improve the pH stability of immobilized enzymes [22].

Optimum temperature is essential to the biotransformation reaction of an enzyme [64]. The ionic states of amino acids forming the active sites of enzymes may be influenced by temperature. The general unfolding of protein structures may occur, leading to the inactivation of the enzymes [22]. The optimum temperatures for free and immobilized tannase were 80 °C and 50 °C, respectively (Figure 5c), which differed from the majority of immobilized enzymes in raising the optimum temperature. The lower optimum temperature reduces the energy consumption of the catalytic process and indicates that immobilization reduces the activation energy of the reaction. We speculate that this is due to the high thermal adaptability of the tannase itself and that the main limiting factor for the catalytic reaction after immobilization shifts to the efficiency of substrate delivery in the enzyme-carrier system. The optimum temperature for immobilized tannase was reduced relative to that of a free enzyme, but immobilization afforded the enzyme with a board temperature range that was 89.8% of that of the relative activity (40–90 °C), probably due to the protective effect of chitosan carrier on the tannase structure [65].

The thermal stability of tannase is critical for its industrial application. Figure 5d–f showed the residual enzyme activities of free and immobilized tannase after treatment at 50 °C, 60 °C, and 70 °C. Free and immobilized tannase retained 55.7% and 58.5% of relative activity, respectively, after 120 h of incubation at 50 °C and retained 34.0% and 57.2%, respectively, after 12 h at 60 °C. However, their activities were gradually lost at high temperatures. Notably, immobilized tannase was slightly less sensitive to high temperatures. The half-life of immobilized tannase at 70 °C was 52.7 min, which was 1.59-fold that of free tannase. The thermal stability of immobilized enzymes usually improves [66,67,68,69] due to their interactions with carriers; these interactions inhibit conformational freedom and the thermal vibrations of peptides [66].

### 2.6. Reusability and Storage Stability of GP–CS–Tannase

**Figure 6:**
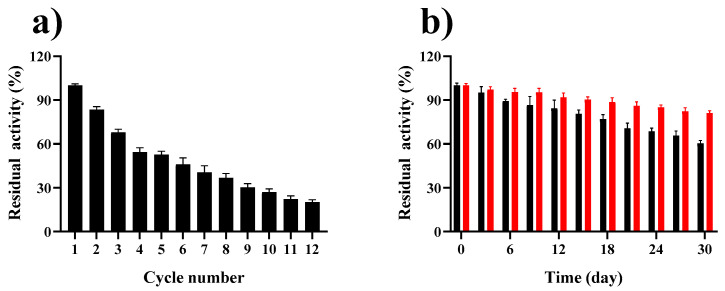
Reusability and storage stability of GP–CS–tannase. (**a**) The reaction was performed at 50 °C for 5 min with 50 mM citrate buffer (pH 6.0). Residual activity was presented as a percentage of initial enzyme activity under experimental conditions. All data represent the mean ± standard deviation of triplicate measurements. (**b**) Tannase (■) and GP–CS–tannase (■) were stored at 4 °C, and their activities were measured under optimal conditions after removal every 3 days. The residual activities of the free and immobilized enzymes were presented as a percentage of initial enzyme activities. All data represent the mean ± standard deviations of triplicate measurements.

The reusability of the enzyme was considered one of the paramount advantages of immobilized enzymes. The reusability of GP–CS–tannase was evaluated by implementing 12 continuous recycles. GP–CS–tannase retained 46.1% of the initial activity after six recycles and retained 20.1% of the initial activity after 12 cycles (Figure 6a). Storage stability is a fundamental criterion for the performance of immobilized biocatalysts for preparative or industrial use. The storage stability of free and immobilized tannase was investigated by measuring residual activity for 30 days (Figure 6b). GP–CS–tannase retained higher (81.12%) residual activity than free tannase (60.37%) throughout the testing period.

### 2.7. Effect of Extraction Temperature and Time on Catechins in Green Tea

**Figure 7:**
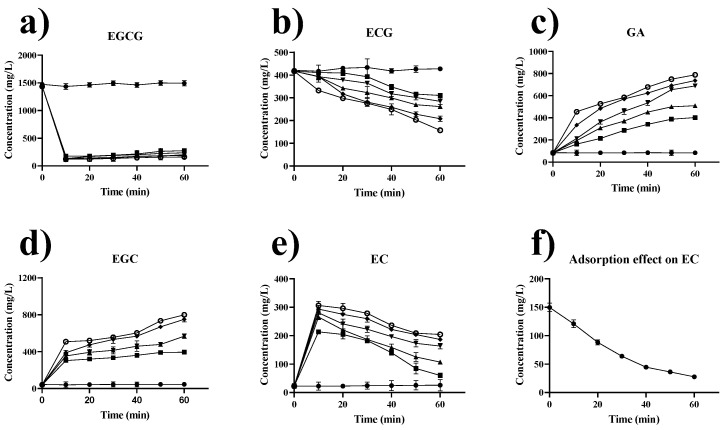
Effect of extraction temperature and time on catechins in green tea. (**a**) EGCG, (**b**) ECG, (**c**) GA, (**d**) EGC, (**e**) EC, and (**f**) adsorption effect on EC. The legend in Figure (**a**–**e**) is as follows: untreated tea extract (●), 30 °C (■), 40 °C (▲), 50 °C (▼), 60 °C (◆), and 70 °C (○); 0.5 g of GP–CS–tannase was added to 10 mL of tea extract, and enzyme activity was 12.5 U. All data represent the mean ± standard deviation of triplicate measurements.

Given the significant loss of immobilized enzyme activity at 80 °C, the tea extract was treated at 30–70 °C for 1 h. Samples were collected at 10 min intervals for the analysis of catechin composition. The results are shown in Figure 7. The conversion rates of ester-type catechins (ECG and EGCG) to non-ester-type catechins (EC and EGC) by tannase increased with temperature (Figure 7a,b). The concentration of GA was also increased with temperature (Figure 7c) due to the accelerated reaction rate. The degradation rate of ECG in tea extracts treated at 70 °C reached 62.7% at 60 min of extraction, exhibiting an enhancement of 36.3% compared with that at 30 °C (Figure 7b). Notably, the degradation rate of EGCG reached more than 95% after 10 min of interaction with tannase at any temperature (Figure 7a). At 70 °C, the concentration of EGC reached 799.4 mg/L, showing an increase of 1758% relative to that of the untreated tea extract (Figure 7d). The concentration of EC reached 306.1 mg/L after 10 min at 70 °C and gradually decreased with further treatment (204.2 mg/L, 60 min) (Figure 7e). We speculated that the possible reason for the decreased EC is the adsorption effect on EC. The adsorption experiment was carried out. The result (Figure 7f) showed that the concentration of the EC standard solution decreased from 149.5 mg/L to 27.5 mg/L due to the adsorption of 122.0 mg/L GP–CS, thereby supporting our hypothesis. The catechins composition analysis indicated that the concentration of non-ester-type catechins, EGC and EC, were increased by 1758% and 807%, respectively, after enzymatic treatment.

### 2.8. Effect of Tannase Treatment on the Biological Activity of Tea Extracts

#### 2.8.1. Effect of Enzymatic Treatment on the Total Phenols of Tea Extracts

**Figure 8:**
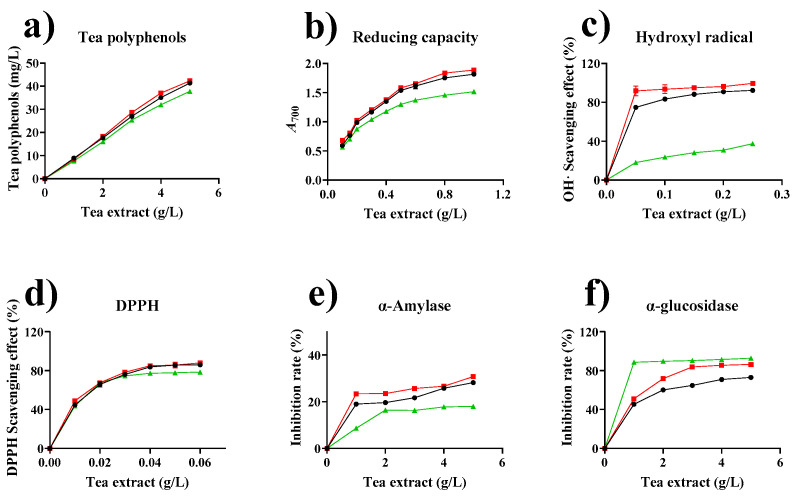
Effect of tannase treatment on the biological activities of tea extract. (**a**) Tea polyphenols. (**b**) Reducing capacity. (**c**) Hydroxyl radical-scavenging effect. (**d**) DPPH·-scavenging effect. (**e**) Inhibition rate of α-amylase. (**f**) Inhibition rate of α-glucosidase. (●), (■), and (▲), represent tannase-treated, GP–CS–tannase-treated, and untreated tea extracts, respectively. All data represent the mean ± standard deviation of triplicate measurements.

Phenol content affects the antioxidant activity of tea extracts. Bursal et al. found that total phenol content was related to iron reduction ability [70]. Ranilla et al. found correlations between phenolic components and antioxidant activity in several traditional medicinal plants, herbs, and spices in Latin America [71]; Castiglioni et al. explored the correlation between phenolics and antioxidant activity in tea by adjusting steeping conditions (time, temperature, and particle size) [72]. As shown in Figure 8a, enzymatic treatment can increase total phenolic content. When the tea extract was added at a volume of 5 g/L, the total phenolic contents in tea extracts treated with free enzymes and immobilized tannase were enhanced by 9.6% and 12.1%, respectively, compared with the total phenolic content of untreated tea extract.

#### 2.8.2. Effect of Enzymatic Treatment on the Reducing Capacities of Tea Extracts

Reduction in Fe^3+^ is an indicator of a phenol’s antioxidant activity [73]. Figure 8b demonstrates that all the tea extracts reduced iron ions, indicating a dose-effect association between tea concentration and reducing capacity. When the tea extract concentration was increased from 0.1 g/L to 1.0 g/L, the optical density (OD) value of immobilized tannase-treated tea extract increased from 0.682 to 1.885, whereas the OD value of free tannase-treated tea extract increased from 0.593 to 1.815. Meanwhile, the OD value of untreated tea extract increased from 0.565 to 1.516. The scavenging activities of tannase-treated tea extracts were higher than those of the untreated tea extracts. The reducing capacity of the immobilized enzyme-treated tea extract was slightly higher than that of the free enzyme group.

#### 2.8.3. Hydroxyl Radical-Scavenging Effect of Tea Extract

Hydroxyl radicals catalyzed by hydrogen peroxide cause lipid peroxidation, massive oxidative protein degradation, and DNA damage and are involved in human pathology and aging [74]. The scavenging effects of different concentrations of tea extracts on hydroxyl radicals are shown in Figure 8c. The scavenging effects of enzyme-treated tea extracts on hydroxyl radicals were significantly higher than those of the non-enzyme-treated tea extracts. When 0.25 g/L tea extract was added, the scavenging capacities of free enzyme, immobilized enzyme, and control group reached 92.3%, 99.5%, and 37.5%, respectively. The enzyme-treated tea extracts significantly improved the scavenging of hydroxyl radicals because of the improved bioactivity of the tea broth after the structural transformation of catechins.

#### 2.8.4. Antioxidant Activity by DPPH Radical Scavenging Assay

DPPH radicals can accept electrons or hydrogen radicals and eventually become stable anti-magnetic molecules, which can be used as substrates for the evaluation of the ability of antioxidants to scavenge free radicals [75]. As shown in Figure 8d, the DPPH radical-scavenging ability of a tea extract increased with the amount of the tea extract. Polyphenols and catechins are closely associated with DPPH free radical scavenging activity. Tannase catalyzes the hydrolysis of ester catechin, which improves the free radical scavenging activities of tea extracts against superoxide anion, hydrogen peroxide, and DPPH [75,76]. The DPPH radical-scavenging ability of the enzyme treated tea extract was slightly higher than that of the untreated group. Tea extracts prepared with free and immobilized tannase showed similar DPPH radicals scavenging abilities. The order of DPPH radical scavenging activities of catechins is EGCG > ECG > EGC > EC [77].

#### 2.8.5. Inhibitory Effect on α-Amylase and α-Glucosidase Activities

The inhibitory activity of the tea extract on α-amylase is shown in Figure 8e. The inhibitory effect of α-amylase increased with tea extract concentration. We found that at a concentration of 5 g/L, the inhibition rate of the tea extract without enzyme treatment was 18.0% for α-amylase, whereas the inhibition rates of the immobilized and free enzyme-treated tea extracts were 30.8% and 28.2%, respectively, which were enhanced by 56.4% and 70.5%, respectively, compared to the inhibition rate of the untreated tea extract. The inhibitory activities of tea extracts against α-amylase is related to the formation of hydrogen bonds between hydroxyl radicals and the catalytic residues of α-amylase and to the formation of conjugated π-systems, which stabilize interactions with active sites [78]. Ester-type catechins (EGCG and ECG) have a high binding affinity for α-amylase active site residues, thus inhibiting α-amylase activity [79]. However, high concentrations of tea polyphenols inhibit α-amylase activity in a non-competitive manner [80]. This finding is consistent with the results obtained in the current experiment. Moreover, ECG and EGCG are weakly correlated with α-amylase inhibitory activity [81].

The inhibitory effects of tea extracts on α-glucosidase activity were investigated using a colorimetric method (Figure 8f). When 1.0 g/L tea extract was added, the scavenging capacities of free enzyme, immobilized enzyme, and control group reached 45.4%, 51.2%, and 88.5% respectively. The enzymatic treatment of tea extract reduced inhibitory effects on α-glucosidase. Moreover, α-glucosidase inhibitory activity was correlated with EGCG and ECG concentrations. Qu et al. reported that EGCG had the strongest inhibitory effect on α-glucosidase activity, followed by ECG [82]. This result is consistent with the catechin composition analysis results, which showed that the treatment of tea extracts with tannase transformed ester-type catechins (EGCG and ECG) into non-ester-type catechins (EGC and EC).

α-Amylase and α-glucosidase are important inhibitory targets for type 2 diabetes. The inhibition of these hydrolytic enzymes can suppress postprandial hyperglycemia for the management of type 2 diabetes. Plant-based extracts are considered promising and effective inhibitors for α-amylase and α-glucosidase [83]. The experimental results showed that the untreated and treated tea extract exhibited certain concentration-dependent inhibitory effects on α-amylase and α-glucosidase. The inhibitory effect of tea extract on amylase were ranked as follows: GP–CS–tannase-treated, free tannase-treated, and untreated tea extracts. The inhibitory effects of tea extracts on glucosidase were ranked as follows: untreated, GP–CS–tannase-treated, and free tannase-treated tea extracts. The inhibitory effect of α-glucosidase was greater than that of α-amylase. This difference in inhibitory effect between α-amylase and α-glucosidase causes undigested carbohydrates to enter the colon, leading to intestinal bacterial fermentation and subsequent intestinal diseases, such as abdominal pain, flatulence, and diarrhea [84]. After enzymatic treatment, the inhibitory effects of the tea extracts on α-amylase considerably improved, whereas the inhibitory effects on α-glucosidase significantly decreased. This decrease may reduce the risk of intestinal diseases.

## 3. Materials and Methods

### 3.1. Materials and Strain

Propyl gallate (PG), rhodanine, tannic acid (TA), and Gallic acid (GA) were purchased from TCI (Shanghai, China). Acetonitrile (HPLC analytical grade) and acetic acid (HPLC analytical grade) were purchased from Sigma Aldrich (Milan, Italy), and 4-nitrophenyl-α-D-glucopyranoside (pNPG), folin, α-glucosidase, epigallocatechin gallate (EGCG), epigallocatechin (EGC), epicatechin gallate (ECG), epicatechin (EC), 1,1-diphenyl-2-picrylhydrazine (DPPH), genipin, and chitosan were purchased from Yuanye Bio-Technology (Shanghai, China).

Tannase from *Aspergillus oryzae* FJ0123 was expressed in *Pichia pastoris* GS115 and stored in the Key Laboratory of Food Microbiology and Enzyme Engineering of Fujian Province (Jimei University, Xiamen, China).

### 3.2. Preparation and Activation of Chitosan Beads

Chitosan beads were prepared and activated with genipin according to a previously report with minor modifications [33]. In brief, a 4% (*w*/*v*) solution of chitosan was first prepared with a 2% solution of acetic acid, then added dropwise to 2 N NaOH for the coagulation.

Chitosan beads were activated with genipin as an activate agent at different conditions, such as concentrations (0.4%, *m*/*v*), activation temperatures (10 °C), activation pH (3), and activation time (3 h), at a speed of 150 rpm/min. After the activation step, excess genipin was washed off with 200 mL of deionized (DI) water and stored at 4 °C until use.

### 3.3. Enzymatic Immobilization

Approximately 10 U of tannase was added to 50 mM phosphate buffer (pH 7.0) with 200 mg of chitosan beads and incubated at 30 °C and 150 rpm/min for 9 h. The carriers were then separated from the solution and washed five times with DI water for the removal noncovalently bonded proteins. The immobilized tannase was stored at 4 °C.

### 3.4. Activity Assay

Tannase activity was determined with a chromogen formation method using gallic acid and rhodanine (2-thioxo-4-ketothiazolidine) [85]. Absorbance was recorded at 520 nm for the experimental and inactivated enzyme groups. Enzyme activity was defined as the amount of enzyme required to catalyze the production of 1 μmol of gallic acid per minute at 30 °C as one unit of enzyme activity.

The immobilization parameters were calculated as follows:

Activity recovery (%) = (Biocatalyst activity (U))/(Intital activity (U)) × 100

Immobilized activity (U/g) = (Biocatalyst activity (U))/(weight (g))

### 3.5. Characterization of CS, GP–CS, and GP–CS–Tannase by FT-IR and TGA

CS, GP–CS, and GP–CS–tannase were completely dried through vacuum freezing and used for FT-IR and TGA analysis. The samples were ground with KBr (1:100 ratio) in a mortar and pestle for 3–4 min and then made into flakes with a hydraulic press. Infrared spectral data were collected with an FTIR spectrometer (Thermo Fisher Nicolet, iS10, Waltham, MA, USA) in a range of 4000–400 cm^−1^ with 16-scan interferogram at 4 cm^−1^ resolution.

TGA was performed using a TG/DSC instrument (TA, SDT Q600, New Castle, DE, USA) under air/N2 flow (60 mL min^−1^); the heating rate was 10 °C min^−1^ and the temperature range was 30–800 °C.

### 3.6. Assessment of Enzymatic Characteristics, Reusability, and Storage Stability

The effect of pH on the activities of the free and immobilized tannase was investigated and measured after reaction pH was adjusted from 5.0 to 11.0. Four different buffers (50 mM) at pH 3.0–10.0, citrate buffer at pH 3.0–6.0, phosphate at pH 6.0–8.0, Tris-HCl buffer at pH 8.0–9.0, and Gly-NaOH buffer at pH 9.0–10.0. Relative activity at the optimum pH for the free and immobilized tannase was taken as 100%. All reactions were performed in triplicate.

To assess the pH stability of the free and immobilized enzymes, we incubated tannase at different pH (3.0–10.0) for 24 h at 4 °C. The residual activities of the enzymes were measured after incubation. The initial activities of the free and immobilized tannase were taken as 100%. All reactions were performed in triplicate.

The effect of temperature on the activities of the free and immobilized tannase was investigated and measured at reaction temperatures of 20–90 °C. Relative activity at optimum temperature for the free and immobilized tannase was taken as 100%. All reactions were performed in triplicate.

To assess the thermal stability of the free and immobilized enzymes, we incubated tannase at different temperatures (50–90 °C) and times. The enzymes were withdrawn periodically for the measurement of their residual activities. The initial activities of the free and immobilized tannase were taken as 100%. All reactions were performed in triplicate.

To assess the reusability of the immobilized tannase, we performed the continuous enzymatic hydrolysis of PG with immobilized tannase. Immobilized enzyme was withdrawn after each reaction with PG for 5 min, washed three times with citrate buffer (50 mM, pH 5.0), and added to a PG solution for the reaction of next cycle. Residual activity was calculated by taking the activity of the first cycle as 100%. All reactions were performed in triplicate.

To assess the storage stability of the immobilized tannase, we stored free and immobilized tannase at 4 °C. The residual activities of the free and immobilized tannase were measured every 3 days. The activity measured on the first day was taken as 100% for the calculation of residual activities. All reactions were performed in triplicate.

### 3.7. Treatment of Tea Extracts with GP–CS–Tannase

Green tea powder (2 g, 40 mesh) was extracted in a tea/water ratio of 1:50 at 100 °C for 20 min, and immediate filtration in a 100 mL volumetric flask was performed. For enzymatic treatment, 0.5 g of GP–CS–tannase (12.5 U) was added to each 10 mL of tea extract. The reaction temperatures (30 °C, 40 °C, 50 °C, 60 °C, and 70 °C) and reaction times (0, 10, 20, 30, 40, 50, and 60 min) were adjusted and the samples were inactivated by boiling and centrifuged for 30 s (12,000 rpm) for HPLC analysis.

### 3.8. Catechin Composition and Total Polyphenol Content Analysis of Tea Extracts

Catechin composition was analyzed using high performance liquid chromatography (HPLC). The treated tea extract samples were filtered using a 0.22 μm filter and then used in HPLC analysis. Catechin composition was determined at 30 °C and 280 nm with an HPLC column (Symmetry C 18, 5 μm, 4.6 mm × 250 mm). Elution was performed by using phases A (0.5% aqueous acetic acid solution) and B (acetonitrile). The elution procedure was as follows: 6.5% B (0 min), 25% B (16 min), 6.5% B (30 min), and 6.5% B (35 min).

The total polyphenol content of the samples was determined using the Folin-Ciocalteu [71] method. Approximately 1 mL of sample was mixed with 1 mL of forintanol color developer and 5 mL of distilled water, and the mixture was incubated for 5 min at room temperature. Then, 3 mL of Na_2_CO_3_ (7.5%) was added for 60 min at room temperature with protection from light. The absorbance of each sample at 725 nm was measured, and the total polyphenol content was calculated according to the standard curve obtained by measuring a polyphenol standard. Gallic acid solution was used as the calibration curve. The result is expressed as mg gallic acid equivalent per L tea extract.

### 3.9. Biological Activity Detection of Green Tea Extract

#### 3.9.1. Determination of Reducing Power

The reducing power of the tea extract was established using a previously described method [4] with minor modifications. Approximately 0.35 mL of phosphate buffer (0.2 mol/L, pH 7.0), 0.15 mL of green tea extract, and 0.35 mL of K_3_Fe(CN)_6_ solution (1%, *m*/*v*) were added to a 1 mL test tube, mixed, and incubated at 50 °C for 20 min. After the solution was rapidly cooled with ice water, 0.35 mL of trichloroacetic acid solution (10%, *m*/*v*) and 0.15 mL of FeCl_3_ solution (0.1%, *m*/*v*) were added and mixed. Absorbance was measured at 700 nm.

#### 3.9.2. Determination of Hydroxyl Radical-Scavenging Capacity

The hydroxyl radical-scavenging activity was measured with the Fenton method [4] as described previously. In brief, 0.2 mL of green tea extract, 0.2 mL of FeSO_4_ (2.25 mM), 0.2 mL of H_2_O_2_ (8.8 mM), and 0.2 mL of salicylic acid ethanol solution (9 mM) were mixed and incubated at 37 °C for 30 min. The absorbance of the reaction mixture was measured at 510 nm. The scavenging capacities of the hydroxyl radicals were calculated as follows:

Scavenging activity (%) = (1 − A1/A0) × 100

where A0 and A1 represent the blank absorbance value (water was used instead of green tea extract) and the absorbance value of the sample, respectively.

#### 3.9.3. Determination of DPPH Radical-Scavenging Activity

DPPH radical-scavenging activity was measured according to a previous method with minor modifications [86]. Approximately 0.5 mL of DPPH–ethanol solution (0.2 mmol/L) and 0.5 mL of green tea extract was mixed and allowed to react for 30 min at room temperature. Absorbance at 517 nm was assayed. DPPH radical-scavenging activity was measured according to the following equation:

Scavenging activity (%) = (1 − A1/A0) × 100

where A0 and A1 represent the blank absorbance value (95% ethanol was used instead of green tea extract) and the absorbance value of the sample, respectively.

#### 3.9.4. Inhibition Effect on α-Amylase

α-Amylase inhibitory effect was measured according to the report of Ranilla et al. [71]. Approximately 0.25 mL of α-amylase (0.05 mg/mL) was mixed with 0.25 mL of green tea extract at 37 °C for 10 min. Then, 0.5 mL of 0.5% (*w*/*w*) starch solution was added. The mixture was incubated at 37 °C for 30 min. After the reaction, 1 mL of DNS (3,5-dinitrosalicylic acid reagent) was added. The mixture was boiled for 10 min, cooled, and diluted to 10 mL with water, and the absorbance was measured at 540 nm. The percentage of α-amylase inhibition was calculated using the following formula:

Inhibition rate (%) = (1 − A1/A0) × 100

where A0 is the absorbance value of the blank (water was used instead of green tea extract) and A1 is the absorbance value of sample.

#### 3.9.5. Inhibition Effect on α-Glucosidase

α-Glucosidase inhibitory effect was measured according to the report of Liu et al. [86]. Approximately 0.05 mL of α-glucosidase solution and 0.05 mL of green tea extract were added to a 96-well plate and incubated at 37 °C for 15 min. Then, 0.02 mL of pNPG substrate solution was added. The mixture was incubated at 37 °C for another 15 min. Absorbance at 410 nm was measured. Inhibition rate according to the following formula:

Inhibition rate (%) = (1 − A1/A0) × 100

where A0 is the absorbance value of blank (phosphate buffer was used instead of green tea extract) and A1 is the absorbance value of sample.

### 3.10. Statistical Analysis

All data represent the mean ± standard deviations of triplicate measurements. One-way analysis of variance (ANOVA) was used in analyzing the statistical significance of differences in each experiment. Data were processed using Origin (version 2019b) and GraphPad Prism (version 8.02).

## 4. Conclusions

We successfully constructed a non-toxic chitosan-based carrier for immobilizing of tannase and improving the biological activities of tea extracts. The preparation process of the immobilization strategy was optimized, and the characteristics of the immobilized enzyme were assessed. We also studied changes in catechin composition after tannase treatment. The results suggested that treating tea extracts with immobilized tannase can enhance their biological activities, including antioxidant activities and inhibitory effects on digestive enzymes. Overall, this study not only expands the application of chitosan in nontoxic immobilized carriers though genipin activation but also provides a novel strategy for the enzymatic treatment of tea extracts and enhancement of their biological activities.

## Supplementary Materials

The following are available online at https://www.mdpi.com/1660-3397/19/3/166/s1, Figure S1: Immobilization yield and enzyme recovery rate of GP–CS–tannase and GA–CS–tannase.
