# Supplementary material for: Chitosan Activated with Genipin: A Nontoxic Natural Carrier for Tannase Immobilization and Its Application in Enhancing Biological Activities of Tea Extract
Source: Mar Drugs. 2021 Mar 19;19(3):166. doi: 10.3390/md19030166 (PMC8003703; doi:10.3390/md19030166)
Supplement: Supplementary file 1 [file marinedrugs-19-00166-s001.pdf]

Supplementary Material

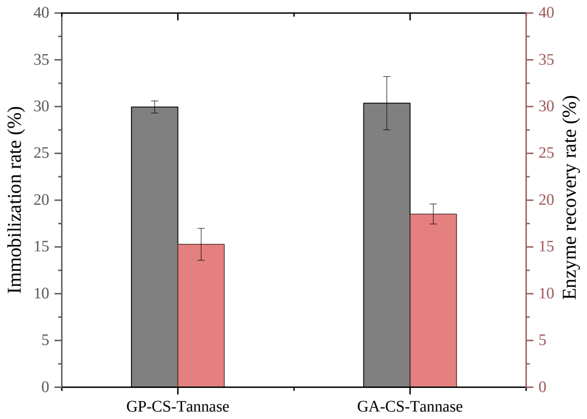

Figure S1. Immobilization rate and enzyme recovery rate of GP-CS-Tannase and GA-CS-Tannase. Activation conditions: genipin concentration (0.4%, m/v), activation temperature (10 °C), activation pH (Citrate buffer at pH 3.0), and activation time (6 h). Immobilization conditions: enzyme amount (10 U), immobilization time (6 h), immobilization temperature (10 °C), immobilization pH (Citrate buffer at pH 5.0), and shaking speed (150 rpm/min)
